# Implant Survival in Immediately Loaded Full-Arch Rehabilitations Following an Anatomical Classification System—A Retrospective Study in 1200 Edentulous Jaws

**DOI:** 10.3390/jcm10215167

**Published:** 2021-11-04

**Authors:** João Manuel Mendez Caramês, Duarte Nuno da Silva Marques, Gonçalo Bartolo Caramês, Helena Cristina Oliveira Francisco, Filipe Araújo Vieira

**Affiliations:** 1 Instituto de Implantologia, Avenida Columbano Bordalo Pinheiro, 1070-064 Lisbon, Portugal; duarte.marques@campus.ul.pt (D.N.d.S.M.); caramesgoncalo@gmail.com (G.B.C.); helenafrancisco@campus.ul.pt (H.C.O.F.); fmaraujo.vieira@gmail.com (F.A.V.); 2 Faculdade de Medicina Dentária, Universidade de Lisboa, 1600-277 Lisbon, Portugal; 3 LIBPhys-FCT UID/FIS/04559/2013, Faculty of Dental Medicine, University of Lisbon, 1600-277 Lisbon, Portugal

**Keywords:** edentulous mandible, edentulous maxilla, implant-supported prosthesis, full-arch, atrophy, retrospective study

## Abstract

This retrospective study analyzed implant survival of immediate implant-supported fixed complete denture (IFCD) treatment options (TOs) based on the level of alveolar atrophy (CC). Records of 882 patients receiving a total of 6042 implants at one private referral clinic between 2004 and 2020 were considered. The mean follow-up period was 3.8 ± 2.7 years. Cumulative implant survival rates (CSRs) were analyzed as a function of CCs and TOs according to Mantel-Haenszel and Mantel-Cox. Hazard risk ratios for implant loss were compared using Cox regression. Confounding factors were identified using mixed Cox regression models. The 2- and 5-year CSRs were 98.2% and 97.9%, respectively. Maxillary 2- and 5-year CSRs were lower (97.7% and 97.3%) compared to mandibular CSRs (99.8% and 98.6%) (*p* = 0.030 and 0.0020, respectively). The CC did not influence CSRs of IFCDs in the mandible (*p* = 0.1483 and 0.3014, respectively) but only in the maxilla (*p* = 0.0147 and 0.0111), where CSRs decreased with increasing atrophy. TOs did not statistically differ in terms of survival rate for a given level of alveolar atrophy. The adaption of IFCD treatments to the level of atrophy and patient-specific risk factors can result in high CSRs, even at different levels of bone atrophy.

## 1. Introduction

Implant-supported fixed complete dentures (IFCDs) represent a well-established treatment modality for edentulism [1,2,3], especially immediately loaded IFCDs have gained increasing popularity among clinicians and patients [4,5,6,7]. Specifically, maxillary and mandibular IFCDs have been shown to result in predictable success rates even in configurations supported by as low as four implants, provided that sufficient primary implant stability can be achieved [8,9,10].

Prolonged edentulism is associated with progressing resorption of alveolar processes [11,12]. This may require adjustment of the implant restoration scheme. As a result, many different treatment options of immediate IFCDs have been suggested [10,13]. Further additional augmentative procedures may be considered, which may impact the long-term clinical prognosis [12,14]. The existing literature evidences that maxillary and mandibular edentulism may be treated successfully using alternative treatment approaches involving four, six, or more implants [15]. However, most of the available scientific literature on fixed rehabilitation of a fully edentulous patient does not establish an association between implant survival rates and the level of bone atrophy.

The importance of diagnostics, treatment planning and choice of an adequate rehabilitation scheme may be supported by different clinical decision support systems (CDSS). Clinical decision support systems (CDSS) can be powerful tools to assist clinical treatment decisions based on patient-specific diagnostic findings [16]. Despite a potential demand, the use of such systems in the dental field has remained low to date [17,18]. Polakovska et al., provided an example of how CDSS may help provide treatment suggestions based on the alveolar anatomic dimensions [19]. Different systems to classify the level of progressing atrophy associated with edentulism have been described in the literature [11,20]. Jensen et al., and Papadimitriou et al., have described the first attempts to use such classifications to select a specific therapeutic scheme for IFCDs [21,22]. A more comprehensive CDSS for IFCDs was recently proposed [23]. This CDSS aims to standardize and propose restorative/regenerative treatment schemes depending on the level of alveolar atrophy from a list of well-established maxillary and mandibular IFCD implant schemes. The system considers a decision process based on the anatomic level and pattern of atrophy of the alveolar process and on patient-specific risk factors to select a specific treatment option from a set of predefined implant rehabilitation and surgical workflow schemes.

To evaluate a possible association between the CSRs of IFCDs and the level of alveolar atrophy, we retrospectively analyzed implant survival in a set of 882 patients treated with immediate IFCDs by applying the mentioned CDSS [23]. Implant survival was analyzed as a function of anatomic classifications and individual treatment options of the CDSS. This study also discusses the potential influence of confounding factors.

## 2. Materials and Methods

### 2.1. Treatment and Follow-Up Regimes

This retrospective study analyzed clinical records of a total of 882 patients that consecutively received routine immediate IFCDs following a recently published CDSS [23]. Treatments were provided in a private referral clinic (Implantology Institute, Lisbon, Portugal) from November 2004 until March 2020 under a certified quality management system and standardized follow-up protocol. The patients were enrolled in a two-weekly recall regime during the first twelve weeks after surgery, followed by regular recalls for professional oral hygiene every 4 months. Postsurgical recall regimes included removing the prosthesis and evaluating the implants at the two-week and twelve-week timepoints and in case of implant or prosthetic complications. Comprehensive medical re-evaluation of the rehabilitation and implant health status were performed every 4 months with prosthesis removal yearly. In addition, the patients were instructed to immediately report any complications or adverse events related to their restoration. Follow-up information was recorded using dedicated software and employed to derive quality indicators for the operation and management of the center.

All the implants were placed conventionally according to the manufacturers’ instructions by a single experienced surgeon (J.M.M.C.). Ancillary procedures like guided bone regeneration or sinus lift procedures were performed according to the predefined schemes of the CDSS [23]. Consistency in pretreatment diagnostics, patient assessment, and patient classification was supported by using identical CBCT device and device settings using a 0.20 mm voxel size, 80 kV, 15 mA, and an exposure time of 12 s (Planmeca Promax, Planmeca, Helsinki, Finland). The patients affected by systemic or local conditions that compromised postoperative healing or osseointegration were excluded from implant treatment.

All the implants were immediately restored with acrylic provisionals and finally restored with a porcelain-veneered zirconia, monolithic zirconia, metallo-ceramic or acrylic-metal hybrid prosthesis. An exception to the immediate loading protocol was found in nine patients involving a total of 21 implants. The sample size of this analysis was a convenience sample determined using patient records displaying adequate diagnostic information with identical presurgical CBCT and fulfilling identical treatment and follow-up criteria.

### 2.2. Definitions

The interval between loading and failure defined the time to implant failure. The implants were considered failed if they presented signs and symptoms that led to implant removal or if the implant was put into sleep [24]. Removed implants comprised the implants that failed due to the lack of osseointegration or due to mechanical failure. Early and late implant failures were defined as failures before or after six months post-placement, respectively [25].

**Figure 1:**
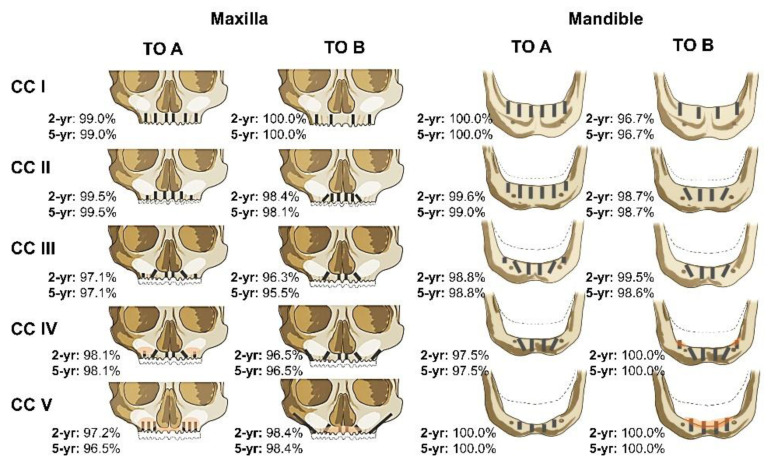
Overview of treatment categories of the applied CDSS with 2-year and 5-year cumulative survival rates (2-yr and 5-yr, respectively). Anatomic categories defining the level of atrophy (CCs) were based on the alveolar dimensions. The CDSS defines three treatment options (TOs), i.e., therapeutic implant configurations, per maxillary and mandibular CC. Only the fixed options, TO A and TO B, are shown for simplicity reasons. Orange regions in CC IV and CC V depict regions with bone augmentation.

The following nominal and categorical factors were considered in the analysis of implant failure:

1. CDSS-related factors, i.e., anatomic category (CCs), treatment options (TOs) and treatment categories (TCs). The applied CDSS defined five different maxillary and mandibular CCs with three TOs per CC [23].
  CCs were defined on a hemi-mandibular treatment unit, i.e., at the quadrant level and based on the vertical and horizontal dimensions at three different predefined positions of the alveolar process from CBCT scans at baseline. TOs (A, B, or C) were defined by the treating clinician based on the planned prosthetic design and under consideration of factors comprising risk factors like systemic conditions, smoking, bruxism, etc., socioeconomic factors, the ability for self-care and oral hygiene as well as the patient’s preferences. Individual TOs defined the characteristics of implant restoration, i.e., number, type, position, and angulation of the placed implant, bone grafting, as well as the type of prosthetic restoration (fixed (A, B) vs. removable (C)). Treatment options as applied as a function of the anatomic classifications are schematically illustrated in Figure 1.
  Treatments deviated from the predefined scheme of the CDSS if considered necessary and included transitions from preexisting restorations under consideration and restoration of preexisting implants. Such preexisting implants were not considered in this analysis.
2. Patient-related factors included gender, age at the time of implant placement, the presence and number of systemic comorbidities including cardiac arrhythmia, arthritis, diabetes type I or II, cardiovascular disease, hepatitis B, HIV, arterial hypertension, hyperthyroidism, osteoporosis and rheumatoid arthritis as well as the self-reported smoking habits and associated daily cigarette consumption.
3. Implantation site-related factors, including jaw type (maxilla, mandible) and jaw location, as categorized into anterior (incisors and canine) or posterior positions (premolar and molar positions).
4. Procedure-related factors: implant system by brand, type, diameter and length and the presence of regenerative bone graft procedures.

### 2.3. Data Collection and Statistical Analysis

A total of 882 patients and 6047 implants were included in the analysis, respectively. Data analysis was carried out in SPPS for statistical analysis (SPPS software, version 24, SPPS Inc., Chicago, IL, USA) by an independent statistician. Descriptive characteristics were reported as the means and standard deviations (SD), medians and interquartile ranges (IQR) and absolute ranges. The differences between descriptive values at the jaw, CC and TO levels were evaluated for statistical significance (*p* < 0.05) using Fisher’s exact test.

CSRs were determined by Kaplan–Meier analysis. Corresponding *p*-values for the comparison of survival curves were calculated using the Mantel–Cox test. Two- and five-year CSR values were statistically compared using the Mantel–Haenszel test. Hazard risk ratios for the implant loss outcome as a function of anatomic classification and treatment option were calculated using Cox regression using the effect of the patient as a random effect. The Firth correction was used when levels had zero events. Confounding factors were derived from individual Cox regression models using one factor as a fixed effect and the effect of the patient as a random effect. Mixed Cox regression models were used to identify the overall risk factors for implant loss after eliminating covariate factors using backward selection of factors that displayed a *p* < 0.20 in the one-to-one associations.

## 3. Results

### 3.1. Descriptive Patient and Study Population-Related Characteristics

The average patient and study population and treatment-related characteristics are summarized in Table 1, Table 2 and Table 3, respectively. In particular, the patients were on average 66.2 ± 11.6 (24–98) years old at intervention and received on average 6.9 ± 2.8 (2–14) implants which were followed up for an average period of 3.8 ± 2.7 (0–14.8) years. Two hundred eight (23.6%) patients were active smokers consuming on average 16.0 ± 7.9 cigarettes per day, affecting 1560 (26%) placed implants. Three hundred seventy-three (42.3%) individuals displayed on average 1.2 ± 0.5 comorbidities affecting 2545 (42%) placed implants. One patient was lost from the cohort due to therapy-unrelated death. Two thousand eight hundred one (46%) implants were placed in the maxilla, 3257 (54%)—in the mandible. Two hundred sixty-one (29.6%) patients received maxillary implants (24.6% (1488) of the implants), 303 (34.4%)—mandibular implants (22.2% (1342) of the implants), 318 (36.1%) patients—bimaxillary reconstructions (53.3% of the implants (3228)). Over the total follow-up period, 111 (1.8%) implants in 86 (9.8%) patients failed after an average loading time of 0.9 ± 1.2 (0.1–7.3) years; 60 (54%) and 51 (46%) implants failed early and late, respectively. Twenty-one (2.4%) patients lost more than one (2–4) implant affecting 46 implants (41% of the failed implants). In 9 (43%) of these patients, 18 (39%) and 19 (43%) implants failed only early or only late, respectively, while only three (14%) patients experienced a combination of early and late failures (nine implants, 20%).

#### 3.1.1. Distribution of Follow-Up Times

Follow-up times for maxillary and mandibular TCs ranged from 2.6 ± 2.1 (IV B) to 4.1 ± 2.6 (II B) years and from 1.4 ± 0.0 (II C) to 8.5 ± 0.4 (V B) years, respectively.

#### 3.1.2. Risk Factor-Related Characteristics

Average patient ages at implantation ranged from 59.2 ± 10.5 (III A) to 69 ± 9.6 (V B) years for maxillary and from 64.6 ± 10.1 (III A) to 74.7 ± 8.2 (V A) years for mandibular treatments, respectively. Differences between individual maxillary and mandibular CCs reached statistical significance (*p* < 0.0001).

Mandibular CC V displayed the highest patient age (73.3 ± 8.3 years). Except for maxillary CC III and V and mandibular CC V, patient age was not statistically significantly different at the TO level. Interestingly, for the mandibular treatment subcohort, an increase in patient age tended to correlate with increasing CC classification and level of atrophy, respectively.

Individuals in the maxillary and mandibular TCs displayed from 0.2 ± 0.4 (I B) to 0.6 ± 0.8 (I A) (*p* = 0.4282) and from 0.0 ± 0.0 (V B) to 0.7 ± 0.8 (III B) (*p* = 0.0983) comorbidities per patient, respectively. Differences did not attain statistical significance at the CC level but were statistically significant at the TO level for maxillary AC III (*p* = 0.0094) and mandibular AC II (*p* = 0.0140), III (*p* = 0.0026) and V (*p* = 0.0320), respectively.

The percentage of smoking individuals per TC ranged from 8% (I B) to 48% (III A) in the maxillary group and between 0% (IV B and V B) and 41% (II A) in the mandibular group. Differences at the CC level reached statistical significance (*p* = 0.0139 and *p* = 0.0002, respectively). Differences between individual TOs reached statistical significance for maxillary AC III (*p* = 0.0154) as well as for mandibular AC II (*p* = 0.0061) and III (*p* = 0.0003).

### 3.2. CDSS-Related Characteristics

The distribution of study characteristics and implant failures as a function of CDSS-related categories and *p*-values as derived using Fisher’s exact test are listed in Table 4.

#### 3.2.1. Characteristics of the CDSS-Related Treatment Provision

**Figure 2:**
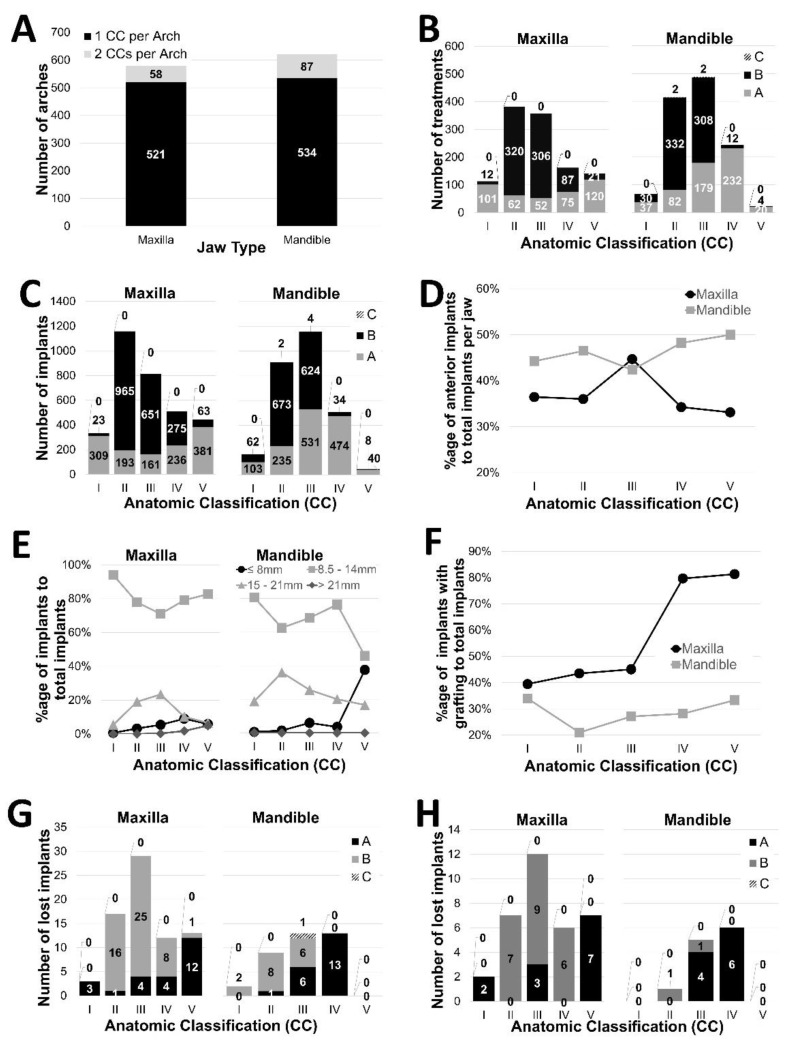
(**A**) Number of different CCs per maxillary and mandibular arch. (**B**,**C**) Total number of treatments (**B**) and implants (**C**) per treatment category. (**D**) Relative %age of anterior implants to total implants per CC. (**E**) Relative distribution of implants per CC as a function of implant length (short implants (≤8 mm), regular implants (8–14 mm), long implants (15–21 mm) and zygomatic implants (>21 mm)). (**F**) Frequency of bone grafting per CC. (**G**,**H**) Total number of failed implants (**H**) and total number of failed implants in patients that lost ≥ two implants (**G**) as a function of TC, respectively.

Treatment provision-related aspects of the CDSS, schematically illustrated in Figure 1, were investigated by analyzing the distribution of study characteristics as a function of CCs and CC/TO combinations as reported in Figure 2 and Table 4, respectively.

As evidenced by the plot in Figure 2A, 521 (90%) and 534 (86%) of the treated maxillary and mandibular arches were classified as symmetric displaying one type of AC in both quadrants.

Treatment and implant numbers were highest in CCs with intermediate bone quantity (CC II and III) accounting together for 740 (64%) and 905 (74%) maxillary and mandibular treatments and 1970 (60%) and 2069 (74%) of the placed implants, respectively (Figure 2B,C).

Maxillary CCs with high and intermediate bone quantity (AC I, II, III) were preferably treated with TOs A (89%), B (84%) and B (85%) (*p* < 0.0001 each), respectively. The cases with limited maxillary bone quantity CC IV were comparably treated with TO A (46%) and B (54%) (*p* = 0.3458), respectively. Treatments of the patients with strongly atrophied maxillae (CC V) were mainly provided as TO A (85%) (*p* < 0.0001). Except for the prominently used TO B in CC II with two implants per quadrant, maxillary restorations were usually provided with 3.0–3.2 implants per quadrant. As further evidenced by Figure 2F, bone grafting in CC IV and V was markedly increased (>80%) compared to CC I–III (<45%). Further, a constant increase in short (<8 mm) and zygomatic implants (>21 mm) in the direction of higher classed CC along with reduced use of long implants (15–21 mm) in CC IV and V was apparent (Figure 2E).

Mandibular treatments were characterized by a trend for configurations with fewer and shorter implants per quadrant in the direction of increasing alveolar atrophy. Specifically, CCs II, III, IV and V displayed a clear shift to one specific TO (*p* < 0.0001 each), i.e., II B (80%), III B (63%), IV A (95%) and V A (83%). Preferred mandibular TOs required 2.0 implants per quadrant on average compared to the corresponding alternative TOs in the corresponding CCs with 2.8–3.0 implants. TOs as part of CC I were comparably distributed between TO A (2.8 implants per quadrant) and B (2.1 implants) (*p* = 0.3924). Further mandibular treatments displayed a higher incidence of anterior implants (45% of the implants) compared to maxillary treatments (38% of the implants) (Figure 2D). Compared to maxillary procedures, mandibular procedures also involved a relatively low percentage of bone grafting (<34%).

Straumann bone level (Straumann Group, Basel, Switzerland) and Zimmer Biomet external hex (Zimmer Biomet, Warsaw, IN, USA) represented the most frequent implant types, with corresponding brands accounting for up to 96% of the placed implants (Table 2). Compared to fixed prostheses (TOs A and B), removable options (TOs C) were only delivered in the total of four mandibular treatments in ACs II and III, respectively.

#### 3.2.2. Implant Loss per Category of the CDSS

The distribution of failed implants in the patients displaying single and multiple (clustered) implant losses is illustrated in Figure 2G,H and reported in Table 4, respectively. A center-weighted distribution of absolute implant loss as a function of CCs that tailed towards higher CCs was identified in the maxilla. In contrast, mandibular absolute implant losses increased from CC I to CC IV. Differences in absolute numbers of lost implants between CCs in both arches were statistically significant (*p* < 0.0001).

Relative implant loss, i.e., the percentage of lost implants compared to the total number of placed implants in the maxilla (2.3%) was statistically higher compared to the mandible (1.3%) (*p* = 0.0106). This parameter also displayed an apparent trend for higher relative failure rates in the higher-ranked CCs (CC IV) in both jaws. Differences between CCs were only statistically significant in the maxilla (*p* = 0.0098) but not in the mandible (*p* = 0.1627) (Table 4).

Except for mandibular CC III (*p* = 0.0044), no significant differences between individual TOs could be identified.

### 3.3. Cumulative Implant Survival Rate and Analysis of Risk Factors Associated with Implant Loss

**Figure 3:**
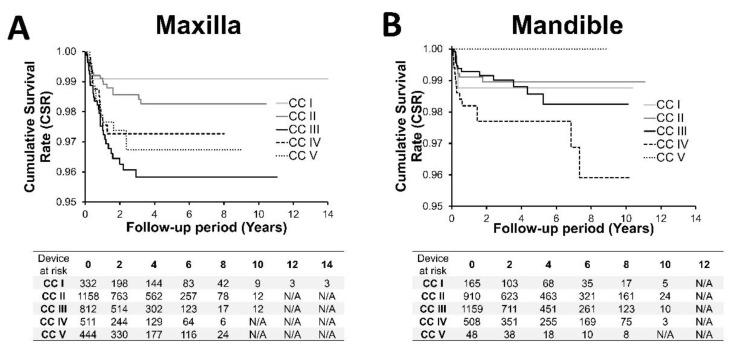
Kaplan–Meier plot, cumulative survival rates for (**A**) maxillary implants and (**B**) mandibular implants as a function of anatomical classifications according to Carames et al., (CCs).

**Figure 4:**
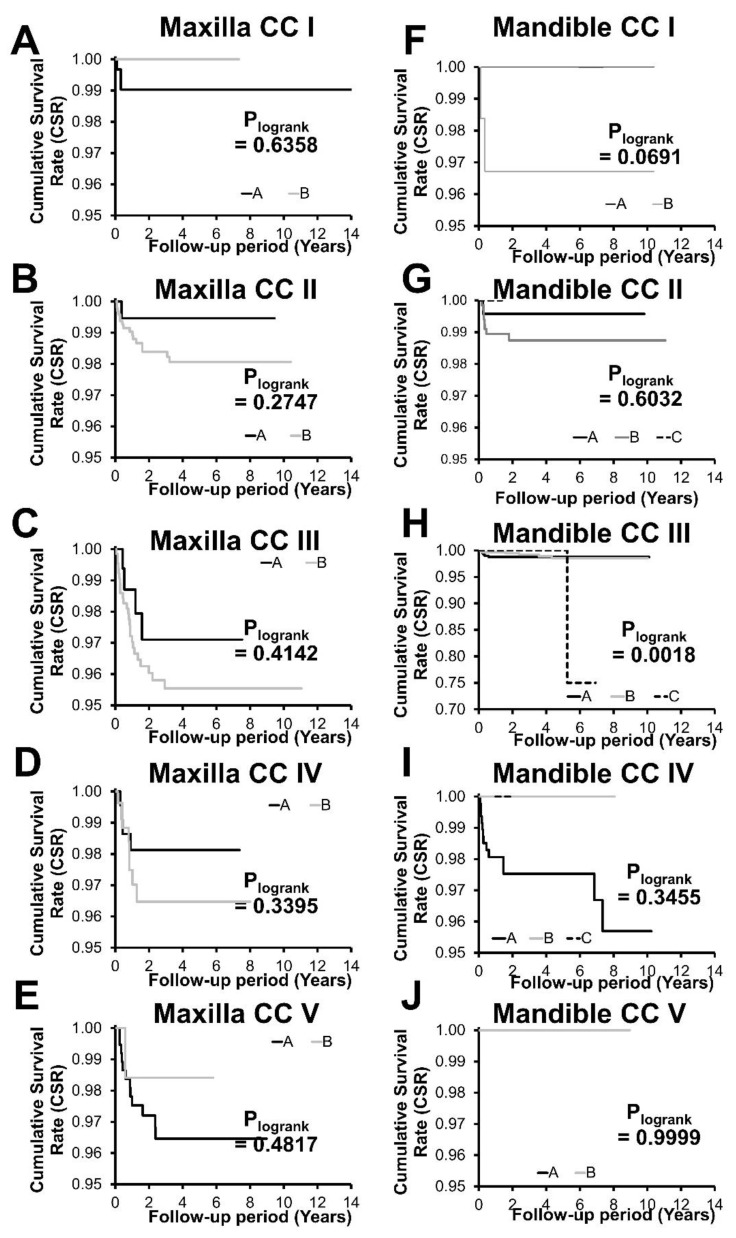
Kaplan–Meier plot, cumulative survival rates of maxillary (**A**–**E**) and mandibular (**F**–**J**) implants placed in individual anatomical classifications (CC I–V, individual graphs) as a function of TOs (**A**–**C**).

Figure 3 and Figure 4 and Table 5 compare the Kaplan–Meier plots and the corresponding 2- and 5-year cumulative implant survival rates (CSR) as a function of CCs and TO, respectively. The total cohort’s overall 2- and 5-year CSRs were 98.2% and 97.9%, respectively. Maxillary CSRs were consistently lower (97.7% and 97.3%) compared to mandibular CSRs (99.8% and 98.6%) after 2 and 5 years, respectively (*p* = 0.030 and 0.0020).

The maxillary implants displayed statistically significant differences at 2 and 5-year CSRs between individual CCs (*p* = 0.0147 and 0.0111, respectively), while differences between the corresponding mandibular CCs failed to reach statistical significance (*p* = 0.1483 and 0.3014, respectively). Overall, CSRs in the maxilla tended to decrease in the direction of CCs with decreasing bone quantity (CC I to V). They ranged from 96.3% (CC III) to 99.1% (CC I) after 2 years and between 95.8% (CC III) and 99.1% (CC I) after 5 years, respectively. Individual values in the mandible ranged from 97.7% (CC IV) to 100% (CC V) and were identical for both endpoints.

At the TO level, none of the TOs within the individual CCs displayed statistically significant differences. Borderline differences were only identified between TO A (100%) and B (96.7%) in mandibular CC I (*p* = 0.0691 for both endpoints).

As reported in Table 6, hazard risk ratios for implant loss (HRR) of the mandibular implants were 0.59 (CI, 0.393–0.884) times lower compared to the maxillary implants (*p* = 0.0106). At the CC level, HRRs were further statistically significantly different between the maxillary CCs (*p* = 0.0441) but not between the mandibular CCs (*p* = 0.2765) (Table 6). Maxillary HHRs tended to increase in the direction of CCs with decreasing bone quantity and were highest for AC III (HRR = 1). CC I (HRR = 0.27, *p* = 0.0397) and CC II (HRR = 0.438, *p* = 0.0118) displayed statistically significantly lower HRRs.

At the mandibular level, no clear trend for the HRR as a function of CC could be identified. CC II displayed the lowest (HRR = 0.834), CC IV—the highest HRR (HRR = 2.104), with CC IV being the only CC displaying borderline significant differences (*p* = 0.0719).

### 3.4. Factors Influencing Implant Loss

The factors influencing the risk of implant loss were analyzed using individual and mixed Cox regression models before and after eliminating covariate factors. As evidenced from the listing in Table 7, the factors CC (*p* < 0.0001), age (*p* = 0.0040), cigarettes per day (*p* = 0.0202), as well as the number of implants (*p* < 0.0001) and implant length (*p* = 0.0004) were identified as the main factors influencing the risk of implant loss.

Specifically, the anatomic classifications III and IV (HRR = 1 and 0.806, respectively) displayed significantly higher risks for implant loss compared to CCs I (HRR = 0.187, *p* = 0.0013), II (HRR = 0.367, *p* = 0.367) and V (HRR = 0.384, *p* = 0.0063). Furthermore, the analysis revealed an increase in the HRR by a factor of 1.026 per year of age increase (*p* = 0.0047), 1.027 per each additional cigarette consumed (*p* = 0.0229), 2.105 per additionally placed implant per jaw (*p* < 0.0001) and 1.072 per mm increase in implant length (*p* = 0.0004).

## 4. Discussion

This retrospective study analyzed implant survival of immediately loaded full-arch reconstructions as part of a recently published clinical decision support system (CDSS). This classification system should be interpreted as an aiding tool for implant-supported full-arch reconstruction and not a clinical decision tree. As part of the analysis, implant survival was analyzed as a function of the degree of osseous atrophy, provided treatment schemes, and potential confounding risk factors. In the context of the classification, it should be considered that corresponding restorative schemes were primarily prosthetically driven and were always based on detailed presurgical digital prosthetic planning. It should further be considered that although the applied CDSS defined TOs for both fixed (TOs A and B) and removable (TO C) restorations, removable options were only provided in a neglectable portion of the analyzed sample (six implants in total). Consequently, the results presented in this analysis primarily apply to fixed implant-supported complete dentures.

To our knowledge, few publications have so far considered larger sample sizes for the retrospective analysis of implant survival of full-arch restorations [26,27]. This study was carried out at a private center focusing on oral rehabilitation with a nationwide referral basis. The long inclusion periods of this study allowed studying implant survival over a wide range of varying parameters, including implant components, surgical and prosthetic protocols, and temporal changes in patient characteristics. However, this aspect also increased the number of potential influencing factors, rendering the analyzed data sample more inhomogenous and its analysis more difficult. Despite these advantages and potential limitations, the study setup supports the extrinsic validity of the sample as being representative of the majority of edentulous patients.

The decision for individual treatment schemes was based on a patient-centered and risk-based approach. The oral health impact profile assesses oral health-related quality of life by a hierarchy of functional and psychological parameters. Patients’ satisfaction and expectations towards an immediate fixed implant prosthesis delivered on the same day of the surgery was the main concern in the rehabilitation of this sample of patients. In a study on patient-centered outcomes of immediate full-arch screw-retained rehabilitations, Dierens et al., reported a significant increase in patient satisfaction and self-reported outcomes such as comfort, function and aesthetics compared to the baseline prior to the treatment [7]. Structured diagnosis and risk assessment represent crucial elements for a patient-centered concept and comprehensive patient-centered treatment plan. This plan requires a structured diagnostic process, and all the patients treated in this sample were treated according to this perspective. Based on this concept, it is understandable that individual alveolar anatomy profiles may require more than one treatment option.

This analysis revealed an overall 5-year CSR of 97.9%. Maxillary values (97.3%) were significantly lower than the corresponding mandibular values (98.6%). Considering that the values in this analysis were obtained over a range of different levels of atrophy, patient conditions and treatment options, they were in good agreement with the corresponding values of 97–97.9% and 98–98.9%, respectively, that were reported as part of recent systematic reviews for comparable follow-up periods [1,15,28,29].

Further, the identified higher CSRs of immediate IFCDs in the mandible compared to the maxilla are also in line with other reports and have been attributed to the reduced bone quality and bone quantity in the edentulous maxilla [28,30,31,32].

To our knowledge, this is the first study that systematically investigated implant survival of immediate IFCDs as a function of the level of alveolar atrophy in a broader patient population. From this analysis, it became apparent that differences between the relative implant loss, CSRs and risk ratios between the maxilla and the mandible were most pronounced under moderately and severely atrophied conditions. This finding raises the question of whether the lower maxillary overall CSR values were associated with treatment provision and risk factor distribution, especially in the higher-ranked CCs (CCs III–V), which calls for a more thorough analysis.

Specifically, the 2- and 5-year mandibular and maxillary CSRs revealed a consistent decrease with an increasing level of atrophy, which reached significant levels only in the maxilla. This observation is in line with the general notion that implant placement in the maxilla is regarded as more challenging compared to the mandible [33]. This aspect may, at the same time, also suggest a potential limitation of the applied classification. Although bone quantity was the main criterion for defining the different levels of the classification system, bone quality (density) was not evaluated. Considering the lower bone density in the maxilla, this factor may help explain the identified differences [30].

Interestingly, in the mandible (except for CC V), CSR values in severely atrophic conditions were distinctly lower when compared to non- or moderately atrophied states. Implant losses and clustered losses in the corresponding CC IV were further mainly and exclusively associated with TO A. Chrcanovic et al., recently attributed such clustering to the presence of specific local and systemic risk factors [31]. Our analysis suggests that clustering is consistently associated with lower CSRs and higher risks for implant loss of specific TCs. However, for the specific mandibular CC IV, no potential bias associated with the disparate distribution of risk factors between the subcohorts TO A and B could be identified, suggesting a potential causal relationship with the corresponding therapeutic schemes. Specifically, TO A was based on four interforaminal implants, while TO B involved two additional distal short implants, resulting in a potentially more favorable anteroposterior load distribution.

Interestingly, mandibular 5-year CSRs tended to be higher or equivalent for alternative treatment options of one CC with six implants when compared to four implants irrespective of the level of mandibular atrophy. Despite this consistent pattern, differences between mandibular TOs failed to reach statistical significance, which is in line with a recently reported systematic analysis on the effect of implant numbers on long-term implant survival [15]. While the placement of four interforaminal implants is generally well-established, the placement of six implants comprising short molar implants has recently been proposed [8,15,34,35]. Such restorations have been reported to prevent posterior cantilevers and potentially reduce marginal bone loss [35,36,37]. On the other hand, sufficient evidence suggests that prosthetic cantilevers may result in successful treatment options for fully edentulous patients with high long-term survival rates [38]. Further in this context, it needs to be considered that possible additional risk factors like parafunctional habits (bruxism) or the nature of the antagonist were not evaluated as part of this analysis, which might be considered a limitation of the performed analysis.

In the maxilla implant survival, relative implant loss and associated risk levels were significantly associated with the level of alveolar atrophy. Specifically, CSRs distinctly decreased when comparing non-atrophic (99%) to moderately and severely atrophic conditions (96–97%). Regarding a potential limiting bias by associated risk factors, a distinct increase in the percentage of active smokers in the direction of the higher-ranked CCs was noted, which appeared to correlate with lower implant survival. This aspect was also reflected by identifying the level of cigarette consumption as a risk factor for implant loss from the mixed Cox regression model. This observation is in line with several systematic reviews that reported a significantly higher risk of implant failure in smoking patients than in non-smokers, with implant failure risk ranging between 1.87 and 2.38 [39,40].

Severe maxillary bone atrophy has been associated with a loss of available bone volume and increased level of sinus pneumatization, which impede implant placement [13]. In our analysis, this was reflected by a markedly increased rate of bone grafting, use of configurations with ≥ six implants and an elevated frequency of short and zygomatic implants in the highly atrophic CCs IV and V. Treatments in the moderately atrophied CC III were by contrast mainly provided as part of an all-on-four type restoration (TO B). This specific TC also involved a relatively moderate rate of bone augmentation, an average of 4.6 implants per jaw and a pronouncedly higher percentage of anterior implants. Interestingly, this specific TC also displayed the lowest CSR values of all the applied TCs, which was slightly lower than the 97.7% reported in the literature for this configuration [41]. For this comparison, however, it must be considered that this implant configuration has exclusively been applied in moderately atrophied conditions (CC III) as part of the applied treatment schemes.

From a biomechanical perspective, maxillary full-arch configurations with distal implants may distribute applied mechanical loads to the molar alveolar crest region more evenly when compared to all-on-four type configurations. However, their realization is often impeded by decreased trabecular bone density in the posterior atrophied maxilla [13,42]. In this context, it is interesting to note that the relative implant loss in the posterior maxilla was higher (2.6%) compared to anterior positions (1.7%) but appeared to be disproportionally high (5.2%) in CC III B when compared to CC III A (2.3%). This observation may suggest that the elevated biomechanical load on posterior implants in the four-implant configuration CC III B might not be adequately compensated by the anatomic osseous conditions of the CC. The specific use of TO B in CC III might need to be balanced against the corresponding alternative TO A to further improve this CC’s CSR.

Implant loss in the severely atrophic maxillary CCs IV and V also displayed marked clustering of implant loss associated with lower CSRs of TO B and A, respectively. TC IV B was based on two distal pterygoid implants and displayed CSR values comparable to the literature-reported value [10,43]. Interestingly, in contrast to previous reports for this configuration, a disparity between anterior and posterior relative implant loss of 1.1% vs. 3.7% was noted [43]. Treatment in the severely atrophic maxillary CC V was mainly provided as TO A with six straight implants after sinus augmentation compared to TO B with zygomatic implants. Both TOs can be considered complex and required horizontal bone augmentation; still relatively high 5-year CSRs of 96.5% and 98.4%, respectively, were observed.

Finally, the risk factor analysis using mixed Cox regression models confirmed a significant and pronounced effect of anatomic classification on implant loss. Interestingly, the highest hazard ratios were reported for CC III, while other CCs with lower or higher levels of atrophy displayed lower hazard ratios. Furthermore, in line with previous reports, smoking habits had a pronounced and strong effect on implant loss [39,44]. This effect was previously associated with the potential alteration of physiological processes related to osseointegration and potential behavioral differences in oral health and maintenance habits between smokers and non-smokers [45,46,47].

Increasing patient age was found to increase implant loss modestly. Chrcanovic recently reported a decreased risk for implant loss with progressing age and related this to a lower incidence of bruxism and decreased masticatory forces in elderly patients [26,31]. By contrast, Sendyk et al., did not identify any significant difference for implant loss between younger and older patient populations, which might explain the weak effect of age on implant loss identified in this study [48]. Lastly, an increased implant length was also identified as a potential risk for implant loss, which may require further investigations.

Clinical studies have indicated that bone grafting can improve CSRs and implant stability in the edentulous atrophied maxilla [14]. As indicated in the treatment schemes in Figure 1, the applied CDSS suggested bone augmentation in the higher atrophied maxillary and mandibular CCs [23]. However, bone augmentation was not identified as a confounding factor affecting implant loss as part of the performed analysis. Hence, any direct influence of bone augmentation in the performed analysis remains speculative. Different bone substitutes may be applied for bone augmentation of the atrophied arches, including autologous bone, allografts or xenografts [49]. Following a patient-centered approach and recent reviews that indicate that CSRs may not be affected by the type of bone graft material used, regenerative procedures performed in the analyzed patient cohort were treated with demineralized bovine bone grafts (DBBM) [50].

Implant survival and success have been associated with biological and technical complications. Specifically, prosthetic screw loosening has been reported to result in bone resorption [51]. In this study, 100% of the implant-supported full-arch reconstructions were screw-retained with multiunit abutments, being particularly relevant as part of the applied treatment regimens to allow for a comprehensive routine medical evaluation of the rehabilitation, specifically regarding the implant status and prosthodontic complications once a year. Related to peri-implant soft tissue health, it is also important to mention that all the implants were immediately restored with multiunit abutments following the one abutment—one time concept. This concept supported undisturbed healing of the surrounding peri-implant mucosal soft tissue to minimize marginal bone loss [52,53].

Future research should consider evaluating this CDSS prospectively. Such research should also consider the patient’s interests and expectations and evaluate patient-reported outcomes like, e.g., health-related quality of life (HRQL) measures before and after an immediate implant-supported fixed complete restoration.

## 5. Conclusions

Based on the analysis of implant survival rates as a function of the level of dimensional alveolar atrophy and applied treatment options presented here and under consideration of the limitations related to its retrospective nature and the lack of control over potential influencing factors, the following conclusions might be drawn:

- The level of alveolar atrophy did not influence implant survival of IFCDs in the mandible, only in the maxilla.
- Implant survival rates in the maxilla tended to decrease with increasing levels of alveolar atrophy and were lower under moderate-to-severe atrophic conditions.
- Individual treatment options did not statistically differ in terms of survival rate for a given level of alveolar atrophy.

In conclusion, adopting the restorative implant scheme to the level of alveolar atrophy and patient risk factors according to the applied clinical decision support system delivered clinically acceptable implant survival rates for all alveolar atrophy levels.

## Figures and Tables

**Table 1:** Average characteristics of the study population. Abbreviations: SD, standard deviation; IQR, interquartile range. Note: ^a^ values relate to the cohort with a diagnosed systemic health condition (*n* = 373), ^b^ values relate to the smoking cohort (*n* = 208), ^c^ values relate to the cohort of patients that lost one or more implants (*n* = 111), ^d^ value corresponds to the implant level (*n* = 6047), ^e^ value includes any replacement implants for the lost implants.

Patient/Implant Characteristics	Mean ± SD	Median (IQR)	Range
Age (years)	66.2 ± 11.6	66.0 (59.0–73.8)	24–98
Number of comorbidities ^a^	1.2 ± 0.5	1 (0–1)	2–5
Cigarettes per day ^b^	16.0 ± 7.9	15.0 (10.0–20.0)	2–40
Placed implants per patient ^e^	6.9 ± 2.8	6.0 (4.0–9.0)	2–14
Lost implants per patient ^c^	1.3 ± 0.6	1.0 (1.0–1.0)	1–4
Follow-up period in years ^d^	3.8 ± 2.7	3.2 (1.4–5.8)	0–14.8
Time to implant loss in years ^c^	0.9 ± 1.2	0.4 (0.3–1.1)	0.1–7.3

**Table 2:** Descriptive patient and cohort-related characteristics. Abbreviations: SD, standard deviation; IQR, interquartile range; N/A, not applicable, i.e., calculation not suitable at the patient or implant level.

Factor	Characteristics	Number of Patients (*n* = 882)	Number of Implants (*n* = 6047)
Gender	Female	560 (63%)	3742 (62%)
	Male	322 (37%)	2305 (38%)
Age	<65	402 (46%)	2889 (48%)
	≥65	480 (54%)	3158 (52%)
Systemic condition	Present	373 (42%)	2545 (42%)
	Absent	509 (58%)	3502 (58%)
Comorbidities	0	509 (58%)	3502 (58%)
	1	298 (34%)	2056 (34%)
	2	64 (7%)	405 (7%)
	3	10 (1%)	72 (1%)
	>3	1 (0%)	12 (0%)
Smoking	Active	208 (24%)	1560 (26%)
	Non-smoker	674 (76%)	4487 (74%)
Procedure type	Maxilla	261 (30%)	1488 (24.6%)
	Mandible	303 (34%)	1334 (22%)
	Bimaxillary	318 (36%)	3225 (53%)
Number of lost implants	0	796 (90%)	N/A
	1	65 (7%)	N/A
	2	18 (2%)	N/A
	3	2 (0%)	N/A
	4	1 (0%)	N/A
Timing of the implant failure	Early	50 (56%)	60 (54%)
	Late	39 (44%)	51 (46%)
Timing of the clustered implant failure (≥two implants)	Early	9 (43%)	N/A
	Late	9 (43%)	N/A
	Early and late	3 (14%)	N/A

**Table 3:** Descriptive treatment and procedure-related characteristics. Abbreviations: SD, standard deviation; IQR, interquartile range; N/A, not applicable, i.e., calculation not suitable at the patient or implant level.

Factor	Characteristics	Number of Patients (*n* = 882)	Number of Implants (*n* = 6047)
Jaw type and location	Anterior maxilla	N/A (–)	1223 (20%)
	Posterior maxilla	N/A (–)	2034 (34%)
	Anterior mandible	N/A (–)	1256 (21%)
	Posterior mandible	N/A (–)	1534 (25%)
Length of the implant	≤8 mm	116 (13%)	274 (5%)
	8–14 mm	854 (97%)	4465 (74%)
	15–21 mm	22 (2%)	1275 (21%)
	>21 mm	17 (2%)	33 (1%)
Regenerative surgery	Yes	320 (36%)	2489 (41%)
	No	562 (64%)	3558 (59%)
Implant system	Straumann	391 (35%)	2365 (39%)
	Zimmer Biomet	643 (58%)	3438 (57%)
	Other brand	81 (7%)	244 (4%)

**Table 4:** Descriptive statistics of study variables stratified by CCs and TOs of the CDSS. Number of treatments (*n* = 2596 treatment units) and number of implants (*n* = 6047) are reported as the total number and percentages relative to individual CCs. Follow-up times are reported at the implant level as the average values and SD. Gender (ratio of female and male patients), patient age (average values and SDs), the number of systemic conditions (average values and SDs) and smoking habits (total number of smokers and relative percentages of smokers in the TC) are reported at the treatment level. Bracketed % values reported at the TO level refer to the % relative to the individual CCs. Bracketed % values reported at the CC levels refer to the % relative to the total value on the maxillary or mandibular level; *p*-values related to differences at the TO and CC level were obtained using Fisher’s exact test; *p*-values indicating statistical significance are marked in bold. Abbreviations: SD, standard deviation.

	Maxilla						Mandible					
	I	II	III	IV	V		I	II	III	IV	V	
**Number of treatments**												
A	101 (89%)	62 (16%)	52 (15%)	75 (46%)	120 (85%)		37 (55%)	82 (20%)	179 (37%)	232 (95%)	20 (83%)	
B	12 (11%)	320 (84%)	306 (85%)	87 (54%)	21 (15%)		30 (45%)	332 (80%)	308 (63%)	12 (5%)	4 (17%)	
C	0 (0%)	0 (0%)	0 (0%)	0 (0%)	0 (0%)		0 (0%)	2 (0%)	2 (0%)	0 (0%)	0 (0%)	
	**<0.0001**	**<0.0001**	**<0.0001**	0.3458	**<0.0001**		0.3924	**<0.0001**	**<0.0001**	**<0.0001**	**0.0011**	
∑	113 (10%)	382 (33%)	358 (31%)	162 (14%)	141 (12%)	**<0.0001**	67 (5%)	416 (34%)	489 (39%)	244 (20%)	24 (2%)	**<0.0001**
**Number of implants**												
A	309 (93%)	193 (17%)	161 (20%)	236 (46%)	381 (86%)		103 (62%)	235 (26%)	531 (46%)	474 (93%)	40 (83%)	
B	23 (7%)	965 (83%)	651 (80%)	275 (54%)	63 (14%)		62 (38%)	673 (74%)	624 (54%)	34 (7%)	8 (17%)	
C	0 (0%)	0 (0%)	0 (0%)	0 (0%)	0 (0%)		0 (0%)	2 (0%)	4 (0%)	0 (0%)	0 (0%)	
*p*	**<0.0001**	**<0.0001**	**<0.0001**	0.0845	**<0.0001**		**0.00141**	**<0.0001**	**0.0044**	**<0.0001**	**<0.0001**	
∑	332 (10%)	1158 (36%)	812 (25%)	511 (16%)	444 (14%)	**<0.0001**	165 (6%)	910 (33%)	1159 (42%)	508 (18%)	48 (2%)	**<0.0001**
**Average number of implants per treatment/quadrant (calculated/theoretical)**												
A	3.1/3	3.1/3	3.1/3	3.1/3	3.2/3		2.8/3	2.9/3	3/3	2/2	2/2	
B	1.9/2	3/3	2.1/2	3.2/3	3/3		2.1/2	2/2	2/2	2.8/3	2/2	
C	N/A	N/A	N/A	N/A	N/A		N/A	1/1 or 2	2/1 or 2	N/A	N/A	
Ø	2.9	3.0	2.3	3.2	3.1		2.5	2.2	2.4	2.1	2.0	
**Follow-up time in years (implant level)**												
A	3.8 ± 3	3 ± 2.7	2.6 ± 1.8	2.9 ± 2.1	4.1 ± 2.6		3.5 ± 2.7	4.4 ± 3.3	3 ± 2.5	4.3 ± 2.9	3 ± 1.5	
B	3.9 ± 2.2	4.1 ± 2.6	3.5 ± 2.3	2.6 ± 2.1	3.2 ± 1.6		4.6 ± 2.9	4.5 ± 2.9	4 ± 2.7	3.9 ± 1.9	8.5 ± 0.4	
C	N/A	N/A	N/A	N/A	N/A		N/A	1.4 ± 0	6.5 ± 0.7	N/A	N/A	
*p*	0.7060	**<0.0001**	**0.0002**	**0.0070**	0.1183		0.0086	0.3091	**<0.0001**	0.8617	**<0.0001**	
ABC	3.8 ± 3	3.9 ± 2.6	3.3 ± 2.3	2.7 ± 2.1	4 ± 2.5	**<0.0001**	3.9 ± 2.9	4.5 ± 3	3.6 ± 2.7	4.3 ± 2.8	3.9 ± 2.5	**<0.0001**
**Gender (female/male patients) (treatment level)**												
A	49/52	34/28	33/19	47/28	91/29		9/28	32/50	86/93	198/34	16/4	
B	8/4	183/137	199/107	58/29	12/9		15/15	186/146	247/61	6/6	4/0	
C	0/0	0/0	0/0	0/0	0/0		0/0	2/0	2/0	0/0	0/0	
*p*	0.3607	0.7800	0.8756	0.6236	0.1073		**0.0410**	**0.0050**	**<0.0001**	**0.0059**	0.9999	
∑	57/56	217/165	232/126	105/57	103/38	**0.0006**	24/43	220/196	335/154	204/40	20/4	**<0.0001**
**Average patient age at treatment (treatment level)**												
A	65.2 ± 11.4	66.2 ± 11.3	59.2 ± 10.5	61.4 ± 10.1	63.3 ± 11.7		65 ± 13.4	66.5 ± 13.8	64.6 ± 10.1	70.7 ± 12.4	74.7 ± 8.2	
B	68.9 ± 17.2	66.3 ± 11	64.9 ± 11.7	60.3 ± 10.7	69 ± 9.6		71.8 ± 11.6	66.3 ± 11	66.7 ± 10.6	66.9 ± 11.3	66.5 ± 1.5	
C	N/A	N/A	N/A	N/A	N/A		N/A	68 ± 0	69 ± 0	N/A	N/A	
*p*	0.9331	0.4286	**0.0027**	0.5552	**0.0143**		0.076965	0.9287	0.1439	0.6028	**0.0125**	
ABC	65.6 ± 12.2	66.3 ± 11.1	64.1 ± 11.8	60.8 ± 10.4	64.1 ± 11.6	**<0.0001**	68 ± 13.2	66.3 ± 11.6	65.9 ± 10.5	70.5 ± 12.4	73.3 ± 8.3	**<0.0001**
**Average number of systemic comorbidities (treatment level)**												
A	0.6 ± 0.8	0.4 ± 0.6	0.3 ± 0.5	0.5 ± 0.9	0.5 ± 0.7		0.4 ± 0.6	0.3 ± 0.6	0.5 ± 0.6	0.6 ± 0.8	0.6 ± 0.5	
B	0.2 ± 0.4	0.5 ± 0.6	0.5 ± 0.7	0.4 ± 0.6	0.6 ± 0.7		0.4 ± 0.7	0.5 ± 0.6	0.7 ± 0.8	0.5 ± 0.5	0 ± 0	
C	N/A	N/A	N/A	N/A	N/A		N/A	1 ± 0	3 ± 0	N/A	N/A	
*p*	0.0547	0.1937	**0.0094**	0.5834	0.6779		0.5216	**0.0140**	**0.0026**	0.8663	**0.0320**	
ABC	0.6 ± 0.8	0.5 ± 0.6	0.5 ± 0.7	0.4 ± 0.8	0.5 ± 0.7	0.4282	0.4 ± 0.7	0.5 ± 0.6	0.6 ± 0.8	0.6 ± 0.7	0.5 ± 0.5	0.0983
**Treatments in smoking individuals (treatment level)**												
A	18 (18%)	17 (27%)	25 (48%)	17 (23%)	38 (32%)		9 (24%)	34 (41%)	58 (32%)	31 (13%)	2 (10%)	
B	1 (8%)	84 (26%)	91 (30%)	24 (28%)	5 (24%)		12 (40%)	82 (25%)	54 (18%)	0 (0%)	0 (0%)	
C	N/A	N/A	N/A	N/A	N/A		N/A	0/2 (0%)	0/2 (0%)	N/A	N/A	
*p*	0.6871	0.8754	**0.0154**	0.5871	0.6101		0.1944	**0.0061**	**0.0003**	0.3723	1.0000	
∑	19 (17%)	101 (26%)	116 (32%)	41 (25%)	43 (30%)	**0.0139**	21 (31%)	116 (28%)	112 (23%)	31 (13%)	2 (8%)	**0.0002**
**Number of failed implants**												
A	3 (100%)	1 (6%)	4 (14%)	4 (33%)	12 (92%)		0 (0%)	1 (11%)	6 (46%)	13 (100%)	0 (N/A)	
B	0 (0%)	16 (94%)	25 (86%)	8 (67%)	1 (8%)		2 (100%)	8 (89%)	6 (46%)	0 (0%)	0 (N/A)	
C	0 (0%)	0 (0%)	0 (0%)	0 (0%)	0 (0%)		0 (0%)	0 (0%)	1 (8%)	0 (0%)	0 (N/A)	
*p*	0.0833	**0.0003**	**0.0001**	0.2482	**0.0023**		0.1573	0.0196	0.7815	**0.0003**	–	
∑	3 (4%)	17 (23%)	29 (39%)	12 (16%)	13 (18%)	**<0.0001**	2 (5%)	9 (24%)	13 (35%)	13 (35%)	0 (0%)	**<0.0001**
**%age of failed implants**												
A	1.0%	0.5%	2.5%	1.7%	3.1%		0.0%	0.4%	1.1%	2.7%	0.0%	
B	0.0%	1.7%	3.8%	2.9%	1.6%		3.2%	1.2%	1.0%	0.0%	0.0%	
C	N/A	N/A	N/A	N/A	N/A		N/A	0.00	25.0%	N/A	N/A	
*p*	0.9999	0.3343	0.4864	0.3991	0.7037		0.1398	0.4649	**0.0440**	0.9999	–	
ABC	0.9%	1.5%	3.6%	2.3%	2.9%	**0.0098**	1.2%	1.0%	1.1%	2.6%	0.0%	0.1627

**Table 5:** Cumulative implant survival rates (CSRs) as derived from the Kaplan–Meier survival analysis. Values refer to individual TOs and CCs (cumulated over individual TOs); *p*-values represent log-rank values; *p*-values indicating statistical significance are marked in bold.

	Maxilla						Mandible					
	I	II	III	IV	V	p	I	II	III	IV	V	*p*
**Cumulative survival rates, 2 years**												
A	99.0%	99.5%	97.1%	98.1%	97.2%		100.0%	99.6%	98.8%	97.5%	100.0%	
B	100.0%	98.4%	96.03%	96.5%	98.4%		96.7%	98.7%	99.5%	100.0%	100.0%	
C	N/A	N/A	N/A	N/A	N/A		N/A	100.0%	100.0%	N/A	N/A	
*p*	0.6358	0.3040	0.4733	0.3395	0.5930		0.0691	0.6032	0.4087	0.3613	0.9999	
∑	99.1%	98.6%	96.3%	97.3%	97.4%	**0.0147**	98.8%	99.0%	99.2%	97.7%	100.0%	0.1483
Total			97.67%						98.81%			**0.0030**
**Cumulative survival rates, 5 years**												
A	99.0%	99.5%	97.1%	98.1%	96.5%		100.0%	99.6%	98.8%	97.5%	100.0%	
B	100.0%	98.1%	95.5%	96.5%	98.4%		96.7%	98.7%	98.6%	100.0%	100.0%	
C	N/A	N/A	N/A	N/A	N/A		N/A	100.0%	100.0%	N/A	N/A	
*p*	0.6358	0.2747	0.4142	0.3395	0.4817		0.0691	0.6032	0.8356	0.3613	0.9999	
∑	99.1%	98.3%	95.8%	97.3%	96.7%	**0.0111**	98.8%	99.0%	98.6%	97.7%	100.0%	0.3014
Total			97.34%						98.60%			**0.0020**

**Table 6:** Calculated risk ratios for the implant loss outcome per anatomic classification (maxillary CCs I–V and mandibular CCs I–V) and individual TOs per CC. The values were calculated from the Cox regression analysis using the effect of the patient as a random effect. The Firth correction was used when levels had zero events. *p*-values indicating statistical significance are marked in bold.

Anatomic Classification (CC)	Treatment Option (TO)	Survived Implants/Lost Implants (% Lost)	Hazard Ratio (95% CI)	*p*-Value	*p*-Value for the Overall Effect
Maxilla	-	3183/74 (2.3%)	1	**0.0106**	**0.0098**
Mandible	-	2753/37 (1.3%)	0.590 (0.393–0.884)		
**Maxilla (CC level)**					
I		239/3 (0.90)	0.27 (0.077–0.940)	**0.0397**	**0.0441**
II		1141/17 (1.47)	0.438 (0.230–0.832)	**0.0118**	
III		783/29 (3.57)	1		
IV		499/12 (2.35)	0.75 (0.361–1.557)	0.4405	
V		431/13 (2.93)	0.782 (0.383–1.592)	0.4982	
**Mandible (CC level)**					
I		163/2 (1.21)	1.109 (0.238–5.153)	0.8948	0.2765
II		901/9 (0.99)	0.834 (0.347–1.998)	0.6832	
III		1146/13 (1.12)	1		
IV		495/13 (2.56)	2.104 (0.935–4.730)	0.0719	
V		48/0 (0.00)	0.839 (0.007–6.306)	0.9059	
**Maxilla (TO level)**					
I	A	306/3 (0.97)	1		0.7096
	B	23/0 (0.0)	1.916 (0.014–19.74)	0.7096	
II	A	192/1 (0.52)	1		0.3444
	B	949/16 (1.66)	2.634 (0.334–20.74)	0.3577	
III	A	157/4 (2.48)	1		0.4383
	B	626/25 (3.84)	1.502 (0.500–4.505)	0.4684	
IV	A	232/4 (1.69)	1		0.3832
	B	267/4 (2.91)	1.667 (0.442–6.276)	0.4501	
V	A	369/12 (3.15)	1		0.4811
	B	62/1 (1.59)	0.498 (0.056–4.384)	0.5302	
**Mandible (TO level)**					
I	A	103/0 (0.00)	1		0.2657
	B	60/2 (3.23)	8.261 (0.672–1139.716)	0.2657	
II	A	234/1 (0.43)	1		0.6342
	B	665/8 (1.19)	2.752 (0.344–22.00)	0.3399	
	C	2/0 (0.00)	42.459 (0.29–804.2432)	0.9265	
III	A	525/6 (1.13)	1		0.095
	B	618/6 (0.96)	0.773 (0.235–2.542)	0.6722	
	C	3/1 (0.25)	14.769 (0.683–319.2)	0.086	
IV	A	461/13 (2.74)	1		0.6813
	B	34/0 (0.00)	0.54 (0.004 –4.088)	0.6813	
V	A	40/0 (0.00)	1		–
	B	8/0 (0.00)	1	–	

**Table 7:** The risk of implant loss as a function of potential risk factors after eliminating covariate factors using a mixed Cox regression model. The factors were reduced by backward elimination if displaying *p* < 0.20 in the one-to-one associations. *p*-values indicating statistical significance are marked in bold.

Risk Factor	Value	Hazard Ratio (95% CI)	*p*-Value	*p*-Value Overall
CCs	I	0.187 (0.067–0.521)	**0.0013**	**<0.0001**
	II	0.367 (0.220–0.610)	**0.0001**	
	III	1		
	IV	0.806 (0.474–1.366)	0.423	
	V	0.384 (0.193–0.763)	**0.0063**	
Age	1 year increase	1.026 (1.007–1.044)	**0.0047**	**0.004**
Cigarettes per day	One cigarette increase	1.027 (1.003–1.051)	**0.0229**	**0.0202**
Implants per jaw	One implant increase	2.105 (1.820–2.433)	**<0.0001**	**<0.0001**
Implant length	One mm increase	1.072 (1.031–1.113)	**0.0004**	**0.0004**

## Data Availability

Data cannot be shared due to data protection obligations.
